# Innovatively Continuous Mass Production Couette-taylor Flow: Pure Inorganic Green-Emitting Cs_4_PbBr_6_ Perovskite Microcrystal for display technology

**DOI:** 10.1038/s41598-018-20376-3

**Published:** 2018-01-31

**Authors:** Young Hyun Song, Seung Hee Choi, Won Kyu Park, Jin Sun Yoo, Seok Bin Kwon, Bong Kyun Kang, Sang Ryul Park, Young Soo Seo, Woo Seok Yang, Dae Ho Yoon

**Affiliations:** 1 Department of Nanotechnology and Advanced Material Engineering, Sejong University, 209 Neungdong-ro, Gwangjin-gu, Seoul, 05006 Republic of Korea; 2 School of Advanced Materials Science and Engineering, Sungkyunkwan University, Suwon, 440–746 Republic of Korea; 3 Electronic Materials and Device Research Center, Korea Electronics Technology Institute, Seongnam, 463–816 Republic of Korea

## Abstract

We report for the first time the mass production of Cs_4_PbBr_6_ perovskite microcrystal with a Couette-Taylor flow reactor in order to enhance the efficiency of the synthesis reaction. We obtained a pure Cs_4_PbBr_6_ perovskite solid within 3 hrs that then realized a high photoluminescence quantum yield (PLQY) of 46%. Furthermore, the Cs_4_PbBr_6_ perovskite microcrystal is applied with red emitting K_2_SiF_6_ phosphor on a blue-emitting InGaN chip, achieving a high-performance luminescence characteristics of 9.79 lm/W, external quantum efficiency (EQE) of 2.9%, and correlated color temperature (CCT) of 2976 K; therefore, this perovskite is expected to be a promising candidate material for applications in optoelectronic devices.

## Introduction

Due to their remarkable photoelectric properties, cesium lead halide perovskites (CsPbX_3_) have recently been widely developed for applications in optoelectronic devices such as displays, light emitting diodes (LED)^1,2^, solar cells^3,4^, photodetectors^5^ and lasers^6,7^.

In particular, the rapid advances in perovskite materials have drawn significant attention to quantum dots (QD) based on the perovskite for use in light-emitting diodes as replacements of conventional QDs such as CdSe/ZnS QDs^8,9^. The QDs based on the CsPbX_3_ perovskite materials were synthesized with the hot injection method and showed narrow emission, high photoluminescence quantum yields over 90%, wide wavelength tunability (400–800 nm) and wide color gamut, thus meeting the requirements of display technology^1,10–12^. Although this optical performance is impressive, there are still challenges that must be overcome for practical use of CsPbX_3_ QDs, such as the realization of large-scale fabrication^13,14^ and stability in moisture when exposed to ambient conditions^15–17^. Therefore, several strategies for not only realization of a high photoluminescence quantum yield (PLQY) but also obtaining stability, such as alloying, passivation, and use of polymer/QD composites have been reported^16–20^.

An alternative fascinating approach to achieve the high PLQY with quantum confinement is to reduce the structural dimensionality of the perovskite^21–23^. The general formula of the perovskite structure is A_n_BX_2+n_, where A is a monovalent cation, B is a divalent metal, and X is a halogen anion. As the n value increases from 1 to 4, the dimensionality of the perovskite structure changes from 3D, to 2D, 1D and 0D, respectively^24,25^. Recently, many studies have reported a remarkable enhancement of PLQY with the change of perovskite dimensionality^26–28^. In the case of the Cs_4_PbBr_6_ solid with the 0D octahedron structure, the PLQY was reported to be 45% higher than that of the 3D structure^28^.

In the LED packaging process, solid state powder is generally more convenient to apply to optoelectronic applications as well as LED package relative to the film form. It is more useful if a fully inorganic Cs_4_PbBr_6_ perovskite solid can be produced on a large scale. Although the synthesis and optical characteristics of Cs_4_PbBr_6_ solid have been reported, to the best of our knowledge, there has been no report on the large-scale production of the Cs_4_PbBr_6_ solid.

Herein, we report for the first time the large-scale synthesis of green-emitting Cs_4_PbBr_6_ perovskite solid as a promising candidate for application in optoelectronic devices via the Couette-Taylor flow method. To date, only mass production of graphene oxide (GO) has been reported within the framework of the Couette-Taylor flow method. The Couette-Taylor flow reactor consists of two coaxial cylinders with inner cylinder rotating and generating toroidal vortices that are regularly spaced along the cylinder axis at the critical rotating speed. This toroidal motion of fluids leads to a highly efficient radial mixing of reactant in the system, thereby enhancing the synthesis reaction efficiency. Fully inorganic Cs_4_PbBr_6_ perovskite powder were used as the luminescence source in the electroluminescence of LEDs. The optimized contents of Cs_4_PbBr_6_ perovskite powder displayed an outstanding PLQY as well as 1931 CIE coordinates for device performance. This technique can be an important method for application in the fabrication of optoelectronic devices.

## Materials and Synthesis

To prepare the Cs_4_PbBr_6_ perovskite powder, all chemicals were used without purification: cesium bromide (CsBr, 99.99%) lead (II) bromide (PbBr_2_, 98%), oleic acid (OA, 65.0–88.0%), oleylamine (OAm, 70%), dimethylformamide (DMF, 99%), hexane (n-hex, 90%), cyclohexane (cyclo-hex, 90%) and toluene (Tol, 99%) were acquired from Sigma Aldrich.

For synthesis of the Cs_4_PbBr_6_ perovskite microcrystals with the Couette-Taylor flow method, 42.562 g of CsBr (0.2 mol) and 73.402 g of PbBr_2_ (0.2 mol) precursor were dissolved in 5 L of DMF. Then, prepared 500 mL of OA and 500 mL OLA were added to 5 L of an n-hexane medium reactor solution. Precursor and medium reactor solutions were added in the Couette-Taylor flow reactor by a peristaltic pump at the same time and same volume (20 mL/min). The Couette-Taylor flow reactor (length: 500 mm) consists of two coaxial cylinders: the outer cylinder (radius: 68 mm) is fixed, and the inner cylinder (radius: 60 mm) rotates. After each solution was introduced into the gap between the two cylinders, the inner cylinder was rotated. The rotating speed of the inner cylinder was 1000 rpm for the reaction time. The mixture in a Couette-Taylor flow reactor produced a green-colored reactant within 15 min. To examine the dependence of the luminous characteristics on the synthetic technique, the stirring method is introduced. 0.085 g of CsBr (0.4 mmol) and 0.146 g of PbBr_2_ (0.4 mmol) were dissolved in 10 mL of DMF. After dissolving the precursor solution, 10 mL was quickly injected into the medium reactor solution, consisting of OA (1 mL) and OAm (1 mL) in 10 mL of n-hexane to induce the reaction via various stirring conditions. The solution color was changed from milky-white to yellow-green. Then, after continuous stirring for 30 minutes, strong green emission was observed under a 365-nm wavelength lamp. The solutions were separated by centrifugation at 4000 rpm for 5 minutes. Then, the supernatant was discarded, and the collected precipitant was washed with 10 mL of cyclohexane. Finally, the precipitant was dried with a freeze-drier to obtain the Cs_4_PbBr_6_ powder. All processes were carried out at room temperature.

### Characterization

The crystalline phase of the Cs_4_PbBr_6_ perovskite was identified using X-ray diffraction (XRD, D-MAX 2500, Rigaku, Tokyo, Japan) with Ni-filtered Cu Kα radiation. The XRD pattern was recorded in the 2θ range of 10°–50°. The photoluminescence (PL) were examined using a spectrometer (SCINCO, FS-2, Korea) in the range of 400–700 nm with a xenon lamp excitation source (150 W). The microstructure and morphology of the samples was investigated using field emission scanning electron microscopy (FE-SEM, JSM-7000F, JEOL). The absolute quantum yield (absQY) was measured using the absolute PL quantum yield measurement system of Hamamatsu C9920–2 and the PL intensity, luminous efficacy, CRI, CCT, CIE coordinate number of the LED operating at 3.2 V and 20, 40, 60, 70 mA were measured using the CSLMS LED 1060 of Labsphere.

## Results

**Figure 1:**
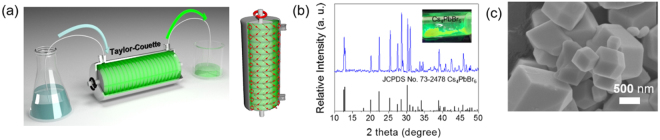
(**a**) Scheme of Synthesis and (**b**) PXRD Patterns (**c**) SEM Image of Cs_4_PbBr_6_ Perovskite Material.

Figure 1(a) shows the procedure for the synthesis of green-emitting Cs_4_PbBr_6_ perovskite materials. PXRD patterns of the green-emitting Cs_4_PbBr_6_ prepared via the Couette-Taylor flow method were obtained. The prepared Cs_4_PbBr_6_ sample shows good agreement with the JCPDS reference No. 73–2478, and no impurity phase is detected; the sample has a crystal structure with a rhombohedral crystal system and lattice parameters of a = b = 13.73 Å and c = 17.31 Å as shown in Fig. 1(b). By using the Couette-Taylor flow method, we confirm the possibility of synthesis of the various perovskite materials such as CsPbBr_3_, Cs_2_PbBr_4_, and Cs_4_PbBr_6_ for innovative mass production. To examine the morphology of the synthesized materials, a field-emission scanning electron microscopy (FE-SEM) image of the synthesized microcrystal using the Couette-Tayler flow method was obtained and is shown in Fig. 1(c). Formation of rhombic prisms is displayed, which corresponds to Cs_4_PbBr_6_ perovskite compounds, as well as previous reports.

**Figure 2:**
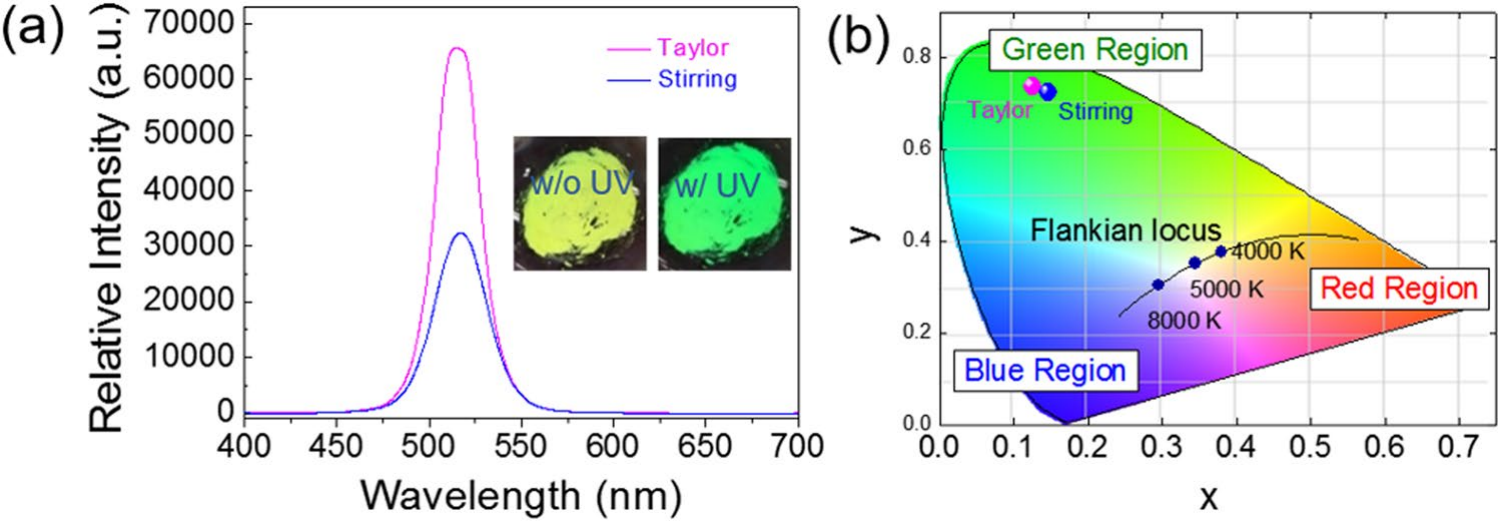
(**a**) Photoluminescence Properties and (**b**) CIE Color Space of Cs_4_PbBr_6_ Perovskite Material.

The photoluminescence (PL) spectra of the Cs_4_PbBr_6_ perovskite microcrystals are shown in Fig. 2(a) as a function of the synthetic route. The PLQY of approximately 46% in the Cs_4_PbBr_6_ microcrystal prepared via the Couette-Taylor flow method displayed the higher luminous performance than the 35% for the sample obtained by the stirring method, and a slight variation of 6.57 nm in the full width at half-maximum (FWHM) was observed. This is related to the decrease in the crystalline defects and the improvement of the homogeneity in the Cs_4_PbBr_6_ microcrystal because the Couette-Taylor flow can intensify the agitation and induce uniform fluidic motion, both of which enhance the uniformity of reactants as well as mass productivity via continuous production. Additionally, the luminous characteristics of the Cs_4_PbBr_6_ perovskite microcrystal are shown ornation as a function of synthesis temperature in Figure S1 of the supporting Inf. Comprehensively consideration of the color purity for display technology shows that the Cs_4_PbBr_6_ perovskite microcrystals synthesized using the Couette-Taylor flow method are suitable for use in display applications. The CIE chromaticity coordinates of the Cs_4_PbBr_6_ perovskite microcrystal is indicated in Fig. 2(b). The CIE color space coordinate of the sample prepared with the Couette Taylor flow method is suited to the green region (x = 0.1254, y = 0.7377), with high color purity compared to the sample obtained using the stirring method (x = 0.1473, y = 0.7241). The overall luminous characteristics as a function of synthetic technique are displayed in Table 1.

**Table 1:** Overall Luminous Characteristics with Synthetic Technique.

Sample	CIE coordinate		FWHM (nm)	PLQY (%)
	X	Y		
Taylor method	0.125	0.737	26.63	46
Stirring method	0.147	0.724	33.2	35

**Figure 3:**
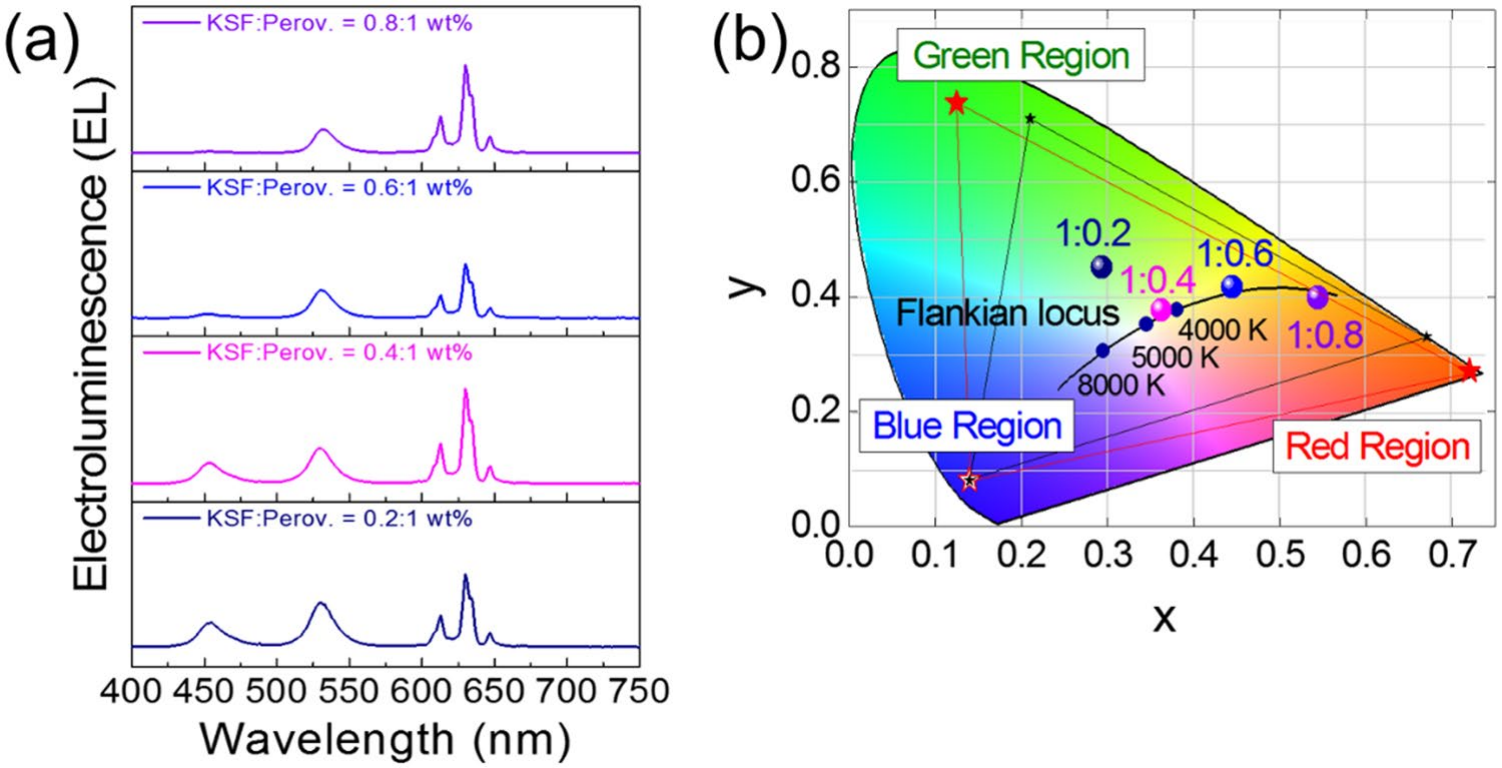
(**a**) Electroluminescence Properties and (**b**) CIE Color Space of Cs_4_PbBr_6_ Perovskite Material for Different Composite Ratios.

**Figure 4:**
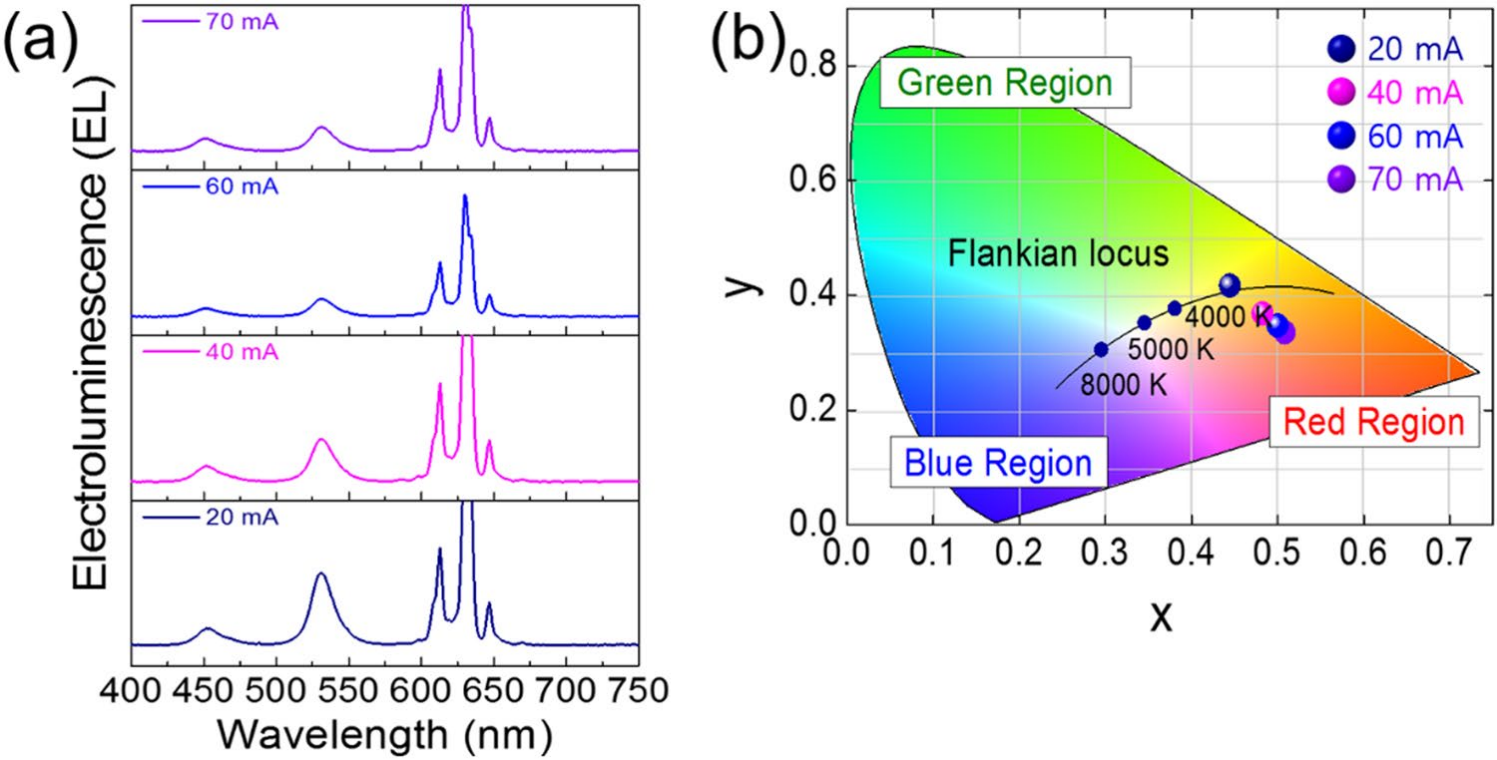
(**a**) Electroluminescence Properties and (**b**) CIE Color Space of Cs_4_PbBr_6_ Perovskite Material for Different Forward-Current Bias.

Electroluminescence properties of a white LED package at the forward-bias current of 20 mA is shown in Fig. 3(a). It consists of a blue-emitting InGaN LED chip, a green-emitting Cs_4_PbBr_6_ perovskite microcrystal, and a red-emitting K_2_SiF_6_:Mn^4+^ phosphor for white light generation. To confirm the change of white target, the K_2_SiF_6_: Mn^4+^ phosphor is composited with Cs_4_PbBr_6_ perovskite microcrystal with the ratio of 0.2:1, 0.4:1, 0.6:1, and 0.8:1, respectively. The typical electroluminescence (EL) spectrum is obtained, corresponding to the emission wavelength of the Cs_4_PbBr_6_ perovskite microcrystal and the K_2_SiF_6_: Mn^4+^ phosphor. Furthermore, luminous efficacy increased with the increasing ratio of the composite. Under the forward-bias current of 20 mA, the highest power efficacies of 9.79 lm W^−1^, CRI of 34.772, CCT of 2976 K, EQE of 2.9% are observed. With an increasing composite ratio, warm-white light is generated due to the addition of the red component. CIE color space coordinates for practical application in white LED are shown in Fig. 3(b). With an increasing composite ratio, the axis is moved to warm white based on the Flankian locus line, which is suitable for use in lighting technology through a perfect combination of green and red components. Additionally, the National Television System Committee (NTSC) has conducted investigations to identify candidates for next-generation display applications, which encompass 118%. To the best of our knowledge, it is very impressive for Cs_4_PbBr_6_ perovskite microcrystal to meet the requirements for use in the ultra-high definition (UHD) display. The overall characteristics of the white LED package are presented in Table 2. Based on the composite ratio of 0.6:1 (K_2_SiF_6_: Cs_4_PbBr_6_), EL spectra and CIE color space coordinates are analyzed as shown in Fig. 4(a,b). We know that perovskite materials are unstable at higher temperatures. With increasing forward-bias current, the luminous efficacy is decreased from 9.79 to 4.48 lm W^−1^, and CCT is changed from 2976 to 1657 K due to the unstable characteristics of the Cs_4_PbBr_6_ perovskite microcrystal. The overall characteristics of the white LED package with the different forward-current bias values are presented in Table 3. Figures S2, S3, and S4 of the Supporting Information show the CIE color space coordinates as a function of the composite ratio under different forward-bias currents of 20, 40, 60 and 70 mA.

**Table 2:** Overall Luminous Characteristics for Different Composite Ratios.

Composition ratio KSF: Perov. (wt%)	Luminous Efficacy (lm/W)	CRI(%)	CCT (K)	CIE coordinate	
				x	y
0.2: 1	7.46	65.345	6563	0.2935	0.4506
0.4: 1	8.87	42.824	4521	0.3623	0.3765
0.6: 1	9.79	34.772	2976	0.4445	0.4165
0.8: 1	7.51	34.577	1777	0.5439	0.3979
20 mA, 2.85 V

**Table 3:** Overall Luminous Characteristics for Different Forward-Current Bias.

Perov.: KSF = 1: 0.6 (wt%)					
Current (mA)	Luminous Efficacy (lm/W)	CRI (%)	CCT (K)	CIE coordinate	
				x	Y
20	9.79	34.772	2976	0.4445	0.4165
40	6.48	30.177	2100	0.4823	0.3681
60	5.08	33.690	1797	0.4999	0.3474
70	4.48	36.642	1657	0.5081	0.3353

## Conclusion

In summary, we have developed an innovative synthetic technique of Cs_4_PbBr_6_ perovskite microcrystal using Couette taylor flow method. The prepared Cs_4_PbBr_6_ perovskite microcrystal shows the behavior of excellent luminous properties with narrow FWHM and mass production possibility. Through these results, we believe that our innovative technique will represent a new strategy for applications in optoelectronic device, and findings are new family member as a next generation candidate in field of luminescence materials^29,30^.

## Electronic supplementary material

Supporting Information
