# Supplementary material for: Innovatively Continuous Mass Production Couette-taylor Flow: Pure Inorganic Green-Emitting Cs4PbBr6 Perovskite Microcrystal for display technology
Source: Sci Rep. 2018 Jan 31;8:2009. doi: 10.1038/s41598-018-20376-3 (PMC5792638; doi:10.1038/s41598-018-20376-3)
Supplement: Supplementary file 1 — Supporting Information [file 41598_2018_20376_MOESM1_ESM.docx]

**Innovatively Continuous Mass Production Couette-taylor Flow: Pure Inorganic Green-Emitting Cs_4_PbBr_6_ Perovskite Microcrystal for display technology**

Young Hyun Song^1,a^, Seung Hee Choi^2,a^, Won Kyu Park^2,a^, Jin Sun Yoo^3^, Seok Bin Kwon^2^, Bong Kyun Kang^2^, Sang Ryul Park^1^, Young Soo Seo^1^, Woo Seok Yang^3,*^ and Dae Ho Yoon^2,*^

^1^Department of Nanotechnology and Advanced Material Engineering, Sejong University, 209 Neungdong-ro, Gwangjin-gu, Seoul 05006, Republic of Korea

^2^School of Advanced Materials Science and Engineering, Sungkyunkwan University, Suwon 440-746, Republic of Korea,

^3^Electronic Materials and Device Research Center, Korea Electronics Technology Institute, Seongnam 463-816, Republic of Korea

^a^These authors contributed equally.

Corresponding author: [dhyoon@skku.edu](mailto:dhyoon@skku.edu) (D.H. Yoon), [wsyang@keti.re.kr](mailto:wsyang@keti.re.kr) (W.S. Yang),

**
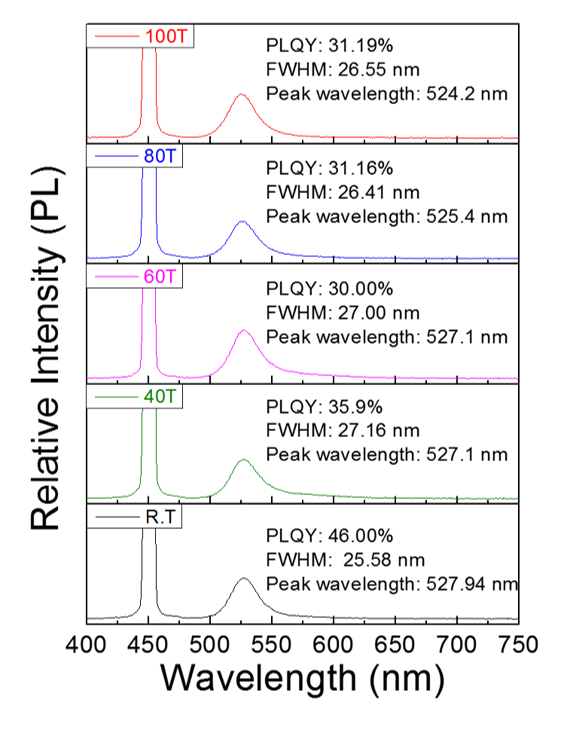
**

Figure S1 of the supporting Information


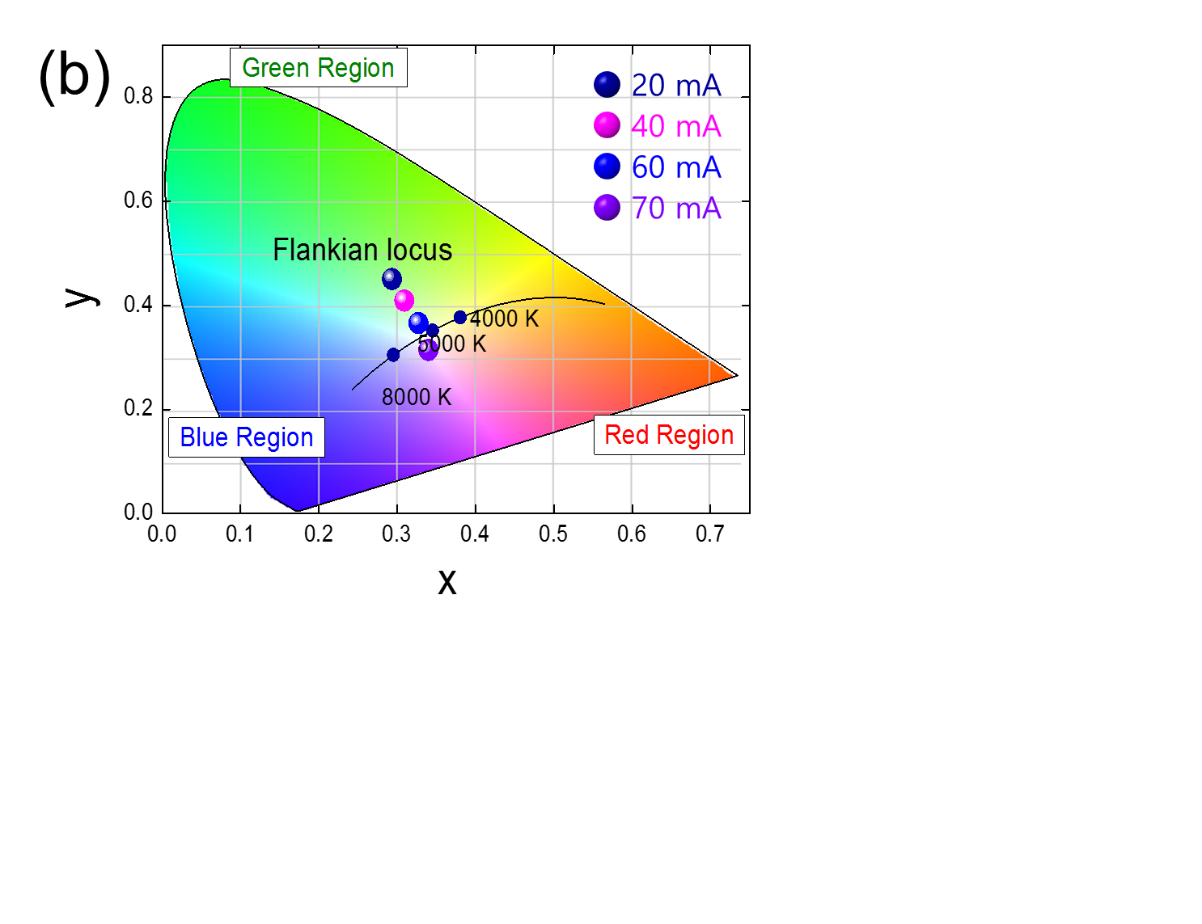

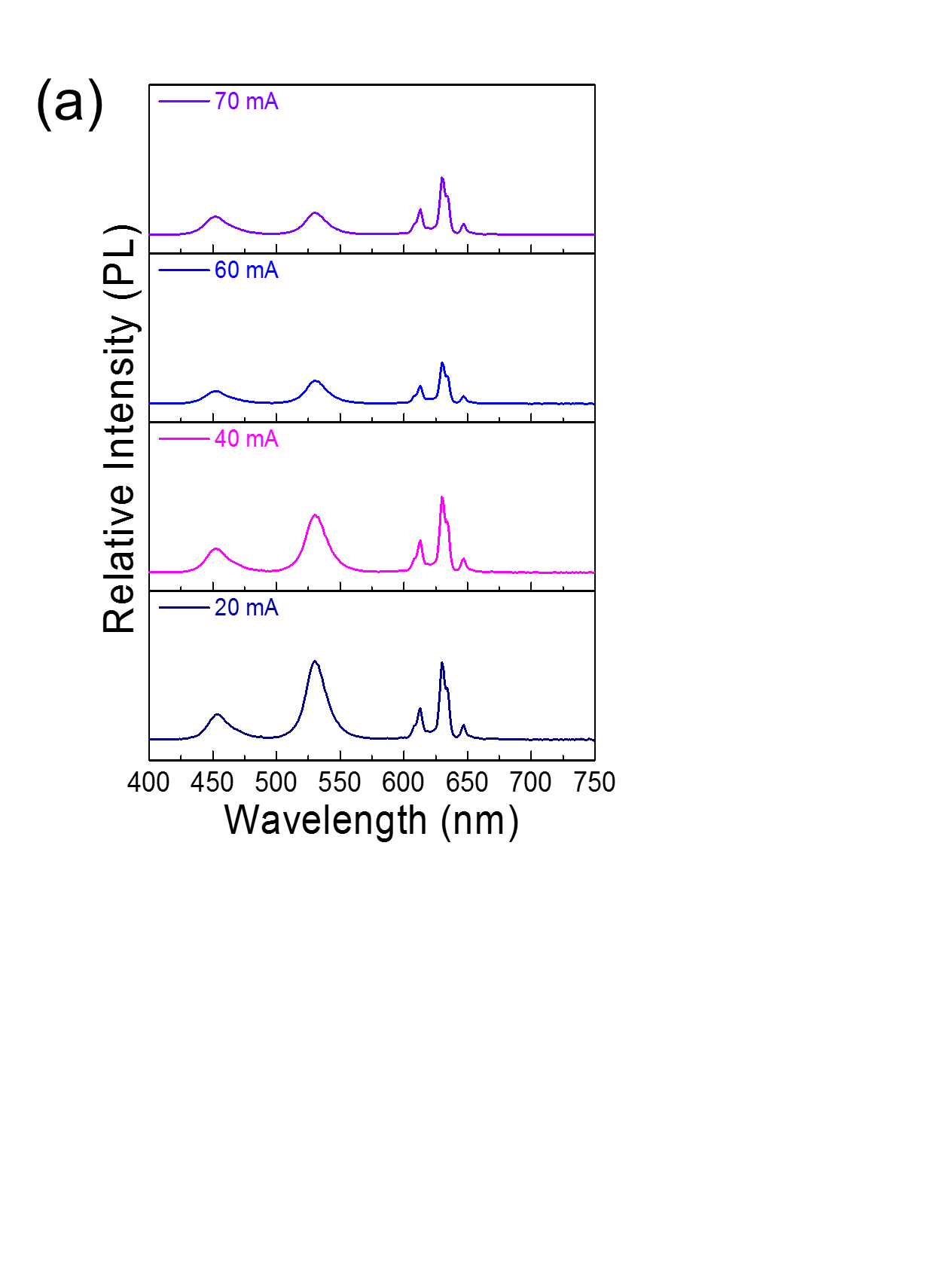


| **Perov. : KSF = 1 : 0.2 (wt%)** | | | | | |
| --- | --- | --- | --- | --- | --- |
| **Bias Current (mA)** | **Luminous Efficacy (lm/W)** | **CRI** **(%)** | **CCT** **(deg. K)** | **CIE coordinate** | |
|  |  |  |  | **x** | **Y** |
| 20 | 7.46 | 65.345 | 6563 | 0.2935 | 0.4506 |
| 40 | 5.17 | 64.224 | 6248 | 0.309 | 0.4094 |
| 60 | 3.62 | 54.489 | 5707 | 0.3271 | 0.3662 |
| 70 | 2.51 | 31.282 | 5095 | 0.3398 | 0.3145 |

Figure S2 of the supporting Information


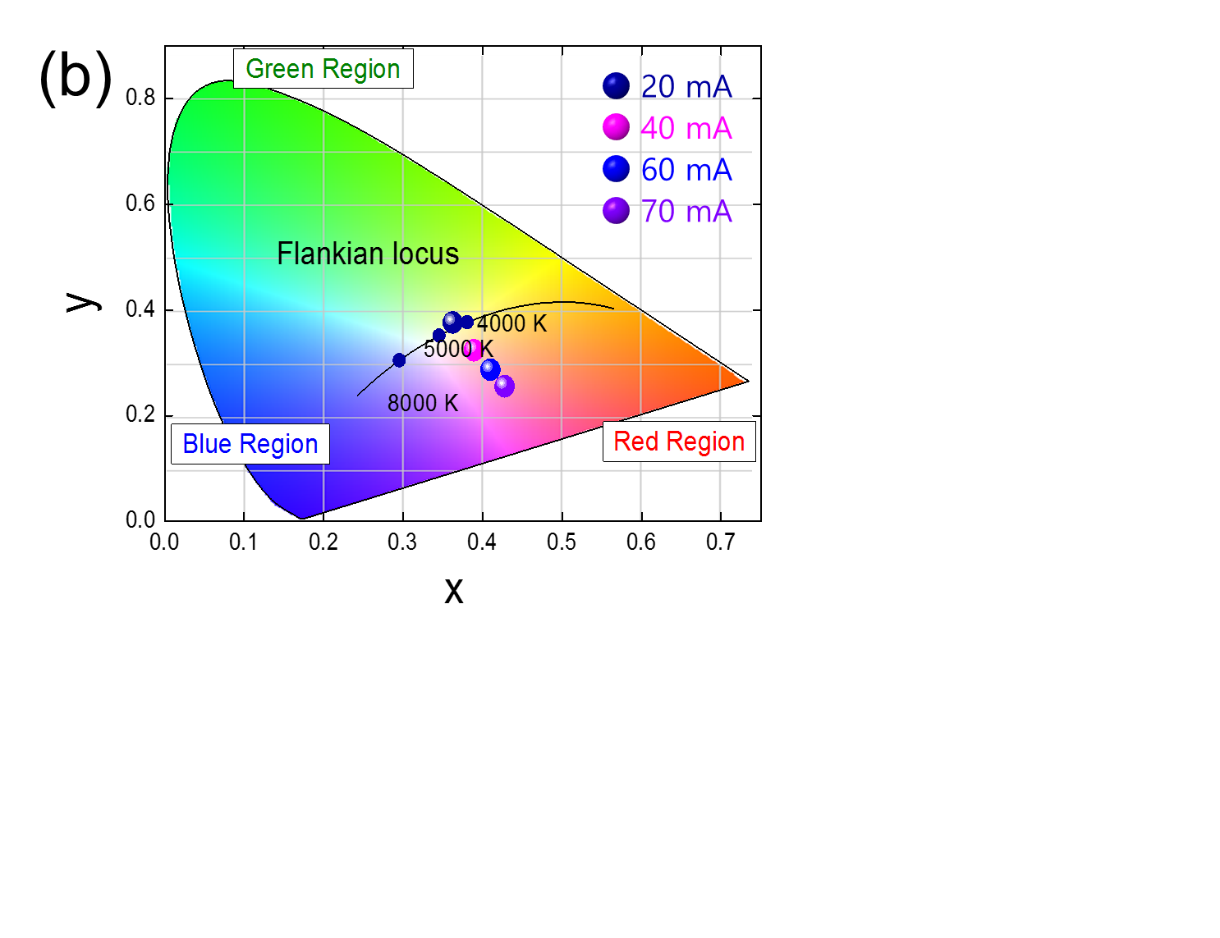

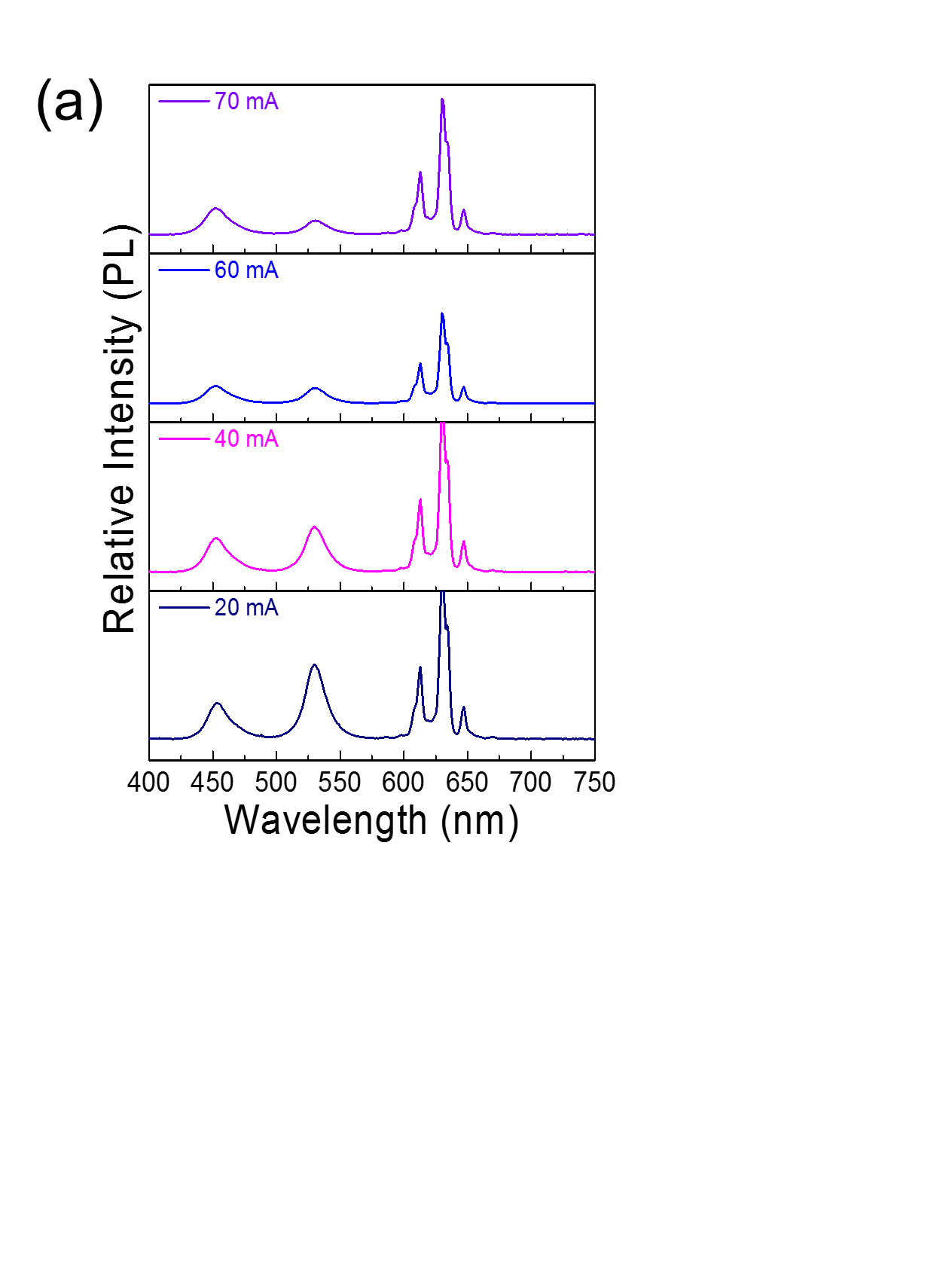


| **Perov. : KSF = 1 : 0.4 (wt%)** | | | | | |
| --- | --- | --- | --- | --- | --- |
| **Bias Current (mA)** | **Luminous Efficacy (lm/W)** | **CRI** **(%)** | **CCT** **(deg. K)** | **CIE coordinate** | |
|  |  |  |  | **x** | **Y** |
| 20 | 8.87 | 42.824 | 4521 | 0.3623 | 0.3765 |
| 40 | 5.73 | 23.089 | 3235 | 0.3891 | 0.3239 |
| 60 | 4.08 | 22.901 | 2316 | 0.4098 | 0.2872 |
| 70 | 3.06 | 21.195 | 1771 | 0.4277 | 0.256 |

Figure S3 of the supporting Information


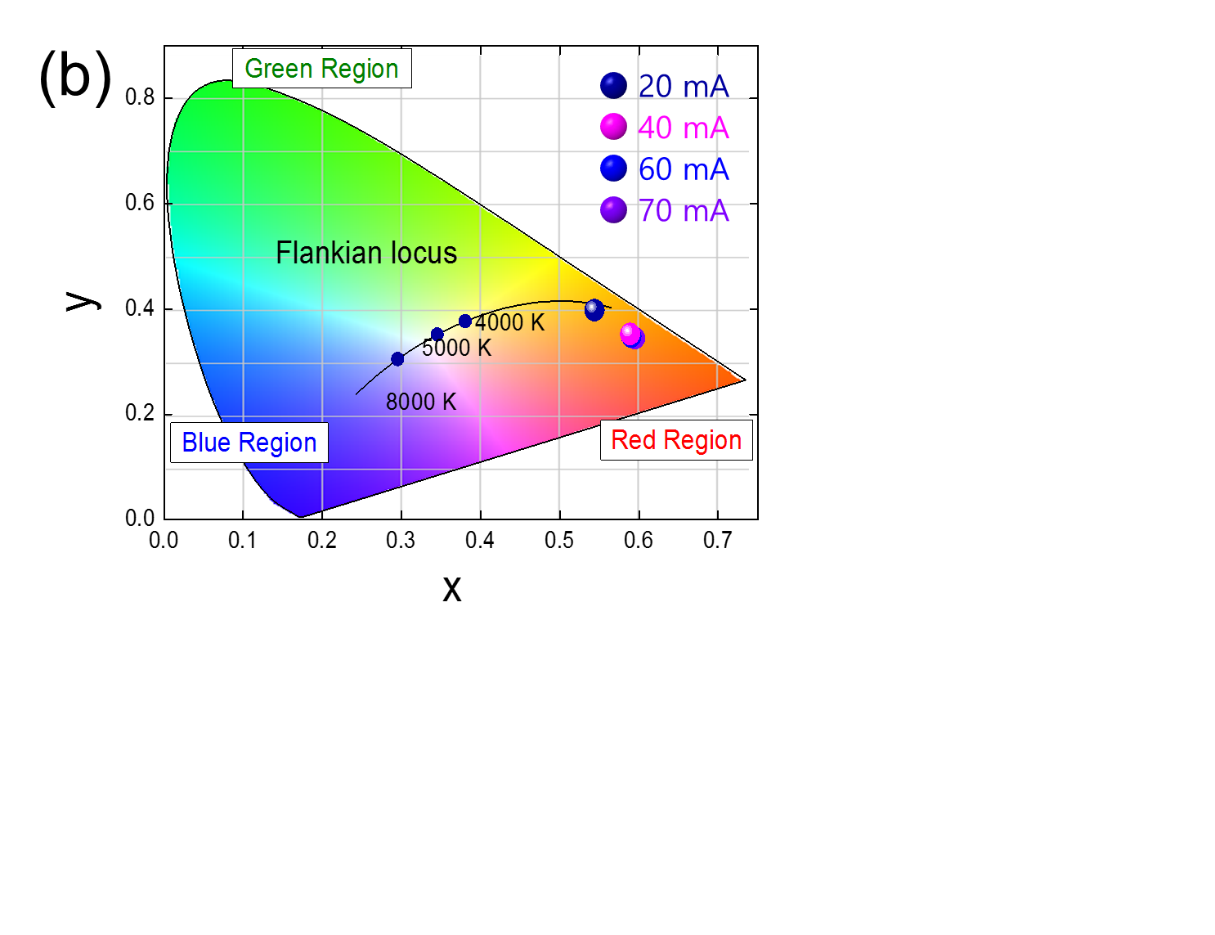

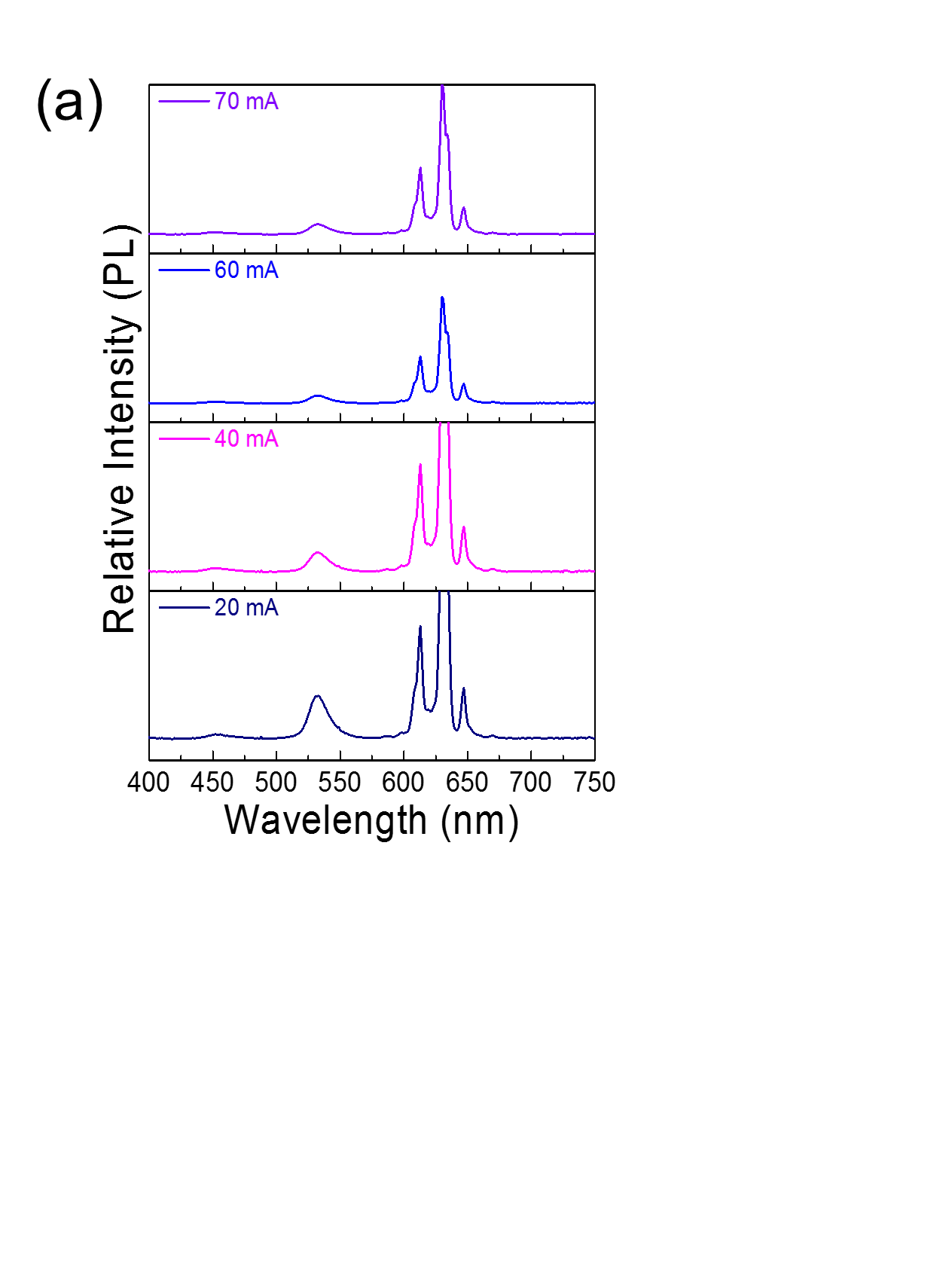


| **Perov. : KSF = 1 : 0.8 (wt%)** | | | | | |
| --- | --- | --- | --- | --- | --- |
| **Bias Current (mA)** | **Luminous Efficacy (lm/W)** | **CRI** **(%)** | **CCT** **(deg. K)** | **CIE coordinate** | |
|  |  |  |  | **x** | **Y** |
| 20 | 7.51 | 34.577 | 1777 | 0.5439 | 0.3979 |
| 40 | 4.75 | 49.885 | 1290 | 0.5888 | 0.3525 |
| 60 | 4.41 | 51.054 | 1262 | 0.5903 | 0.3479 |
| 70 | 4.26 | 52.060 | 1227 | 0.5949 | 0.3448 |

Figure S4 of the supporting Information
